# Melting the wall: plant parasitism entails pectin modification

**DOI:** 10.1080/15592324.2023.2252219

**Published:** 2023-08-29

**Authors:** Sameer Dixit, Santosh Kumar Upadhyay

**Affiliations:** a National Institute of Plant Genome Research, New Delhi, India; b Department of Botany, Panjab University, Chandigarh, India

**Keywords:** Arabidopsis, haustoria, parasitism, PME, PMEI

## Abstract

*Phtheirospermum japonicum* shows induced expression of *PjPME* and *PjPMEI* genes during haustoria development in rice and Arabidopsis with increased PME activity, which leads to the modulated cell wall during parasitism. Moreover, how PME and PMEI proteins interact and balance during haustoria development remains elusive.

Plant parasitism causes serious agricultural losses globally. Parasitic traits in angiospermic plants convergently evolved roughly 12–13 times independently. Thus in common, all parasitic plants form haustoria to develop vascular connections with the host for the absorption of nutrients (Figure 1a). The haustorium development in parasitic plants like *Phtheirospermum japonicum* initiates with the leucine-rich-repeat receptor-like kinase-mediated perception of a suitable haustorium-inducing factor (HIF) such as 2,6-Dimethoxybenzoquinone.^2^ The interaction induces auxin signaling that leads to cell expansion and division, thereby the formation of pre-haustorium.^3^ Then, various cell-wall modifying enzymes secreted by haustorium slack the host cell wall and intrusive cells (ICs) leading to the formation of a xylem connection^4^ (Figure 1a).

**Figure 1.**
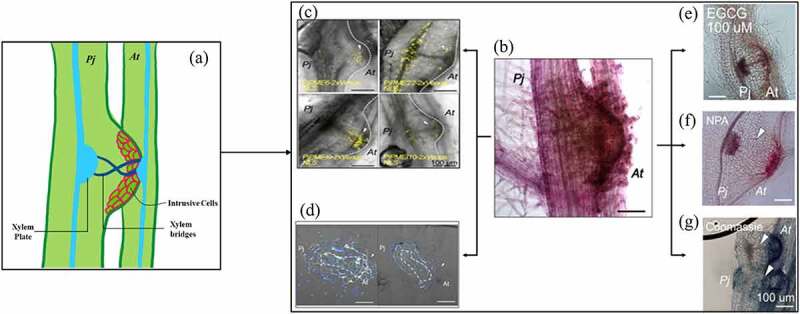
Pectin modification is required for plant parasitism. [a] parasitic plants form haustoria to develop vascular connections with the host, various cell-wall modifying enzymes secreted by haustorium slack the host cell wall and intrusive cells leading to the formation of a xylem bridge. Pectin methylesterase (PME) activity increases during haustoria development [b] due to the tissue-specific expression of *PjPMEs and PjPMEIs* [c] resulting in a dynamic and tissue-specific modification of pectin methylesterification [d]. This induced activity is inhibited by the treatment of epigallocatechin gallate (EGCG), inhibitor of PME [e], *N*-1-naphthylphthalamidic acid (NPA), inhibitor of auxin transport [f], and coomassie brilliant blue, an inhibitor of xyloglucan endotransglucosylase/hydrolase [g] resulted into less haustoria number or xylem bridges deformation. The size of the scale bar is 100 µM in each microscopic figure. The figure is adapted and modified from an open-access article by Leso et al., (2023)^1^ that permits unrestricted use.

The cell wall functions as the first line of defense, therefore cell-wall modifying enzymes play a dynamic role to establish positive interaction under multiple biotic stresses such as viruses, bacteria, fungi, nematodes, and insects. The majority of angiosperms consist of type-I cell walls having ~ 35% pectin, which is made up of methyl-esterified homogalacturonan modified by various enzymes including pectin methylesterases (*PMEs*) and PME inhibitors (*PMEIs*).^5^ Thus, the alteration in pectin composition is a prerequisite for intrusion in the host tissue. Earlier, a few studies reported *PMEs* and *PMEIs* mediated modification in the host cell wall to facilitate invasion by nematodes, fungi and parasitic plants,^6,7^ but the underlying mechanism remains elusive. Like other parasitism, plant parasitism is classified as obligate and facultative on the basis of host dependencies. Plant parasitism is also classified on the basis of the location where the parasitic plant latches onto the host i.e. stem-, and root-parasitic plants. *P. japonicum* is a facultative root parasitic plant of the Orobanchaceae family that parasitizes many plants including *Arabidopsis* and rice, thus providing an excellent model system to study the molecular mechanism of plant parasitism.^2,3^ In a recent study, Leso et al. revealed the mechanism of haustoria development and parasitism by *P. japonicum*.^1^ The authors identified a total of 60 *PjPMEs* and 62 *PjPMEIs* using available proteome data^8^ and revealed their homology with the *AtPME* and *AtPMEI* sequences of the host plant *Arabidopsis thaliana*. The expression profiling using the transcriptome data of time-course infection at the haustorium development site of *A. thaliana*, and intrusive cells and non-intrusive cells of rice revealed increased expression of several *PjPMEs* and *PjPMEIs* in both plants. In addition, a few host *PMEs* and *PMEIs* also showed modulated expression in which an *AtPME* (*AT1G23200*) and *AtPMEI* (*AT2G01610*) exhibited consistent upregulation at several time points. The ruthenium red staining reveals that PME activity increases during early as well as later stages of host invasion and xylem differentiation in *A. thaliana* (Figure 1b). The immuno-histochemical staining with LM19 and LM20 antibodies, which are specific for de-methylesterified and highly methylesterified pectin, also confirmed a similar pattern suggesting dynamic and tissue-specific modification in pectin methylesterification. To validate the specific function, the authors selected three *PjPMEs* (*PjPME6, PjPME22*, and *PjPME51*) and three *PjPMEIs* (*PjPMEI9, PjPMEI10* and *PjPMEI16*) that showed increased expression in both the host plants for further study. Transcriptional reporters assay revealed localization of *PjPME6* and *PjPME51* in ICs, and *PjPME22* in vasculature. However, the results revealed the expression of *PjPMEI9* in ICs and cambium-like tissues, and *PjPMEI10* in plate xylem and ICs. Moreover, *PjPMEI6* reporter did not show any signal (Figure 1). Further, none of these genes showed expression in hairy root tips. The results indicate the specific role of these genes in haustoria development.

How *PME* activity is necessary for haustoria development? To answer this question, the authors performed chemical inhibition of PME activity using epigallocatechin gallate (EGCG) and developed *PMEs* and *PMEIs* overexpression (OE) lines for detailed analyses. The EGCG treatment resulted in reduced haustoria counting and late development of xylem bridge, along with suppressed expression of *PjPMEs* and *PjPMEIs* (Figure 1e). The overexpression of *PjPME6* and *PjPME51* in hairy roots of *P. japonicum* does not affect haustorium induction and development. Nevertheless, *PjPMEI6, PjPMEI9* and *PjPMEI10* overexpression extensively reduced haustoria formation, but not affected xylem connections. The authors also analyzed the impact of highly methylestrified pectin in the host cell wall at haustoria induction using *PMEI5* OE line of *A. thaliana*,^9^ and established the significance of parasitic PME activity in haustoria induction and development.

An earlier study reported a compensatory role of brassinosteroid (BR) signaling in cell wall homeostasis, especially during inconsistency in pectin modification. Any kind of interference in PME activity triggers the BR signaling to protect cell wall integrity^9^. To investigate the role of BR in haustoria development and pectin methylesterification during infection, Leso et al. performed epibrassinolide (epiBL) treatment followed by immunohistochemical staining. External application of epiBL not only decreased haustoria formation, but also reduced the expression of above selected *PjPMEs* and *PjPMEIs*.^1^ Additionally, LM19 and LM20 antibodies-based immunohistochemical staining of haustoria showed reduced accumulation of both unmethylesterified and highly methylesterified pectins after epiBL treatment. Interestingly, *P. japonicum* could efficiently infect *BRl1-EMS-SUPPRESSOR 1* mutants (bes1–2 and bes1-D) having modified brassinosteroid signaling. These findings indicate BR signaling is not essential for haustoria development during infection. Moreover, it is involved in the transcriptional regulation of pectin methylesterification, and therefore it might be associated with cell wall modifications in the parasite.

The co-expression of cambium markers (*PjWOX4* and *PjHB8*) and xylem-markers (*PjCESA7*, *PjVND7* and *PjXCP2*)^10^ with various *PjPMEs* and *PjPMEIs* indicated their role in xylem bridge formation. Further, 1-naphthaleneacetic acid (NAA, a synthetic auxin) treatment could not affect haustoria number and xylem bridge formation. But *N*-1-naphthylphthalamidic acid (NPA, auxin transport inhibitor) reduced xylem bridge formation^3^ as well as de-methylated and highly methylated pectin at the host-parasite interface (Figure 1f). Treatment with coomassie brilliant blue, which inhibits the activity of xyloglucan endotransglucosylase/hydrolase,^11^ increases haustoria count but reduces xylem bridge formation (Figure 1g). The reduced expression of *PjPME51* and *PjPMEI9* during the above treatment suggested their specific role in xylem bridge formation.

Parasitic plants are one of the worst agricultural pests, reducing agricultural productivity and costing billions of dollars globally each year. This study provides insight into the role of *PMEs* and *PMEIs* in haustoria development during plant parasitism and opens a new possibility to achieve resistance by modulating the cell wall esterification through developing *PMEs* or *PMEIs* expressing transgenic plants. Based on this study, one can also develop *PMEs* or *PMEIs* specific inhibitors to regulate plant parasitism. This study provides valuable information but still many questions remain to be addressed. How do *PME* and *PMEI* proteins interact and balance during haustoria development? Do both of them play a significant role during plant parasitism? Or the role of *PMEs* is adequate for haustoria formation and parasitism, while *PMEIs* are only involved in balancing the reaction. Then, what are the other factors controlling the expression of these genes? How does BR-signaling in host plants regulate pectin methylesterification in parasitic plants? The specific role of each *PME* and *PMEI*, and the mechanism of their regulation during host-parasite interaction needs to be addressed in future studies.
